# The Global Burden of Community-Acquired Pneumonia in Adults, Encompassing Invasive Pneumococcal Disease and the Prevalence of Its Associated Cardiovascular Events, with a Focus on Pneumolysin and Macrolide Antibiotics in Pathogenesis and Therapy

**DOI:** 10.3390/ijms241311038

**Published:** 2023-07-03

**Authors:** Ronald Anderson, Charles Feldman

**Affiliations:** 1 Department of Immunology, Faculty of Health Sciences, University of Pretoria, Pretoria 0001, South Africa; 2 Department of Internal Medicine, Faculty of Health Sciences, University of the Witwatersrand Medical School, 7 York Road, Johannesburg 2193, South Africa; charles.feldman@wits.ac.za

**Keywords:** community-acquired pneumonia, cardiovascular events, dendritic cells, macrolides, macrophages, mannose receptor C-type1, platelets, pneumolysin, pro-inflammatory cytokines, *Streptococcus pneumoniae*

## Abstract

Despite innovative advances in anti-infective therapies and vaccine development technologies, community-acquired pneumonia (CAP) remains the most persistent cause of infection-related mortality globally. Confronting the ongoing threat posed by *Streptococcus pneumoniae* (the pneumococcus), the most common bacterial cause of CAP, particularly to the non-immune elderly, remains challenging due to the propensity of the elderly to develop invasive pneumococcal disease (IPD), together with the predilection of the pathogen for the heart. The resultant development of often fatal cardiovascular events (CVEs), particularly during the first seven days of acute infection, is now recognized as a relatively common complication of IPD. The current review represents an update on the prevalence and types of CVEs associated with acute bacterial CAP, particularly IPD. In addition, it is focused on recent insights into the involvement of the pneumococcal pore-forming toxin, pneumolysin (Ply), in subverting host immune defenses, particularly the protective functions of the alveolar macrophage during early-stage disease. This, in turn, enables extra-pulmonary dissemination of the pathogen, leading to cardiac invasion, cardiotoxicity and myocardial dysfunction. The review concludes with an overview of the current status of macrolide antibiotics in the treatment of bacterial CAP in general, as well as severe pneumococcal CAP, including a consideration of the mechanisms by which these agents inhibit the production of Ply by macrolide-resistant strains of the pathogen.

## 1. Introduction: Burden of Community-Acquired Pneumonia (CAP)

Community-acquired pneumonia (CAP) remains the leading infectious disease cause of death worldwide, with considerable impact on the healthcare system; furthermore, despite several advances in diagnosis, management, and prevention of disease, there remains several unmet needs with regard to this infection [1]. While there appears to be a substantial decline in the proportion of *Streptococcus pneumoniae* microorganisms causing CAP in North America, the proportion of this microorganism as a cause in Europe, and other areas of the world, remains much higher, possibly related, it has been said, to differences in childhood pneumococcal conjugate vaccine use (e.g., type of vaccine used (e.g., PCV10 versus PCV13), recommended dosing schedules, total childhood vaccination coverage rates), rates of adult pneumococcal and influenza vaccination, different smoking habits, and possibly, differences in the accessibility to early antibiotic therapy in the different regions [2,3,4]. Nevertheless, in a more recent systematic review of CAP in adults, the most common causative pathogens were noted to be *S. pneumoniae* (pneumococcus) and *Haemophilus influenzae* [3]. However, the authors did note that with increasing use of polymerase chain reaction (PCR) testing for diagnosis, the number of cases of documented causes of CAP has increased, particularly among the viruses, such that the proportions of all bacteria have diminished [3]. A global burden of disease study on lower respiratory tract infections (LRTIs) indicated that in 2016, a total of 2,377,697 (2,145,584–2,512,809) deaths occurred from LRTIs in people of all ages, with 1,080,958 deaths (943,749–1,170,638) in adults over 70 years of age [5]. *Streptococcus pneumoniae* was the leading cause of LRTI morbidity and mortality globally, causing more deaths than all other etiologies combined in 2016 (*Haemophilus influenzae* type b, influenza virus, and respiratory syncytial virus). Progress has been made in reducing the burden of LRTIs, but this has not been equal among all regions and more effort is required, particularly in the case of the elderly. While the prevalence of invasive pneumococcal disease (IPD) was noted to decrease worldwide during 2020 and early 2021, thought to be due to the introduction of non-pharmaceutical interventions to prevent spread of the COVID-19 pandemic, some countries noted IPD levels returning to, and even exceeding, seasonal levels later in 2021 [6,7,8].

## 2. Cardiovascular Complications in Patients with CAP

Considerable research has been undertaken in the past nearly two decades into the occurrence of cardiovascular events (CVEs) in patients with infection, particularly in cases with community-acquired pneumonia, and more recently, COVID-19. Early studies suggested that incident cardiac events in patients with CAP were common, that they were associated with increased short-term mortality, and that older age, nursing home residence, pre-existing cardiovascular disease, and pneumonia severity were factors associated with their occurrence [9]. A more recent systematic review and meta-analysis of observational studies documenting selected CVE in patients with CAP indicated that cardiac complications occurred in 13.9% (95% CI 6.7–12.2), acute coronary syndromes (ACS) in 4.5% (95% CI 2.9–6.5), heart failure in 9.2% (95% CI 6.7–12.2), arrhythmias in 7.2% (95% CI 5.6–9.0), and stroke in 0.71% (95% CI 0.1–3.9) [10]. After adjusting for confounders, CVEs occurring in patients with CAP were independently associated with increased risk for short-term mortality. In a study from the FADOHCECAP Study Group [11], 23.8% of the 1266 patients with CAP who were enrolled experienced at least one CVE.

## 3. Cardiovascular Events in Patients with Pneumococcal CAP

The first study specifically investigating the association between acute cardiac events and pneumococcal pneumonia indicated that this infection was a significant risk for acute cardiac events, such as myocardial infarction, serious arrythmia, or new or worsening congestive heart failure, which substantially increased mortality [12]. Subsequent studies indicated the following: (i) in hospitalized patients with CAP and subsequent CVEs, ACS was more common in those with pneumococcal versus non-pneumococcal CAP, but there was no difference in rates of arrythmias, heart failure, or 1-year mortality [13]; (ii) in a study assessing whether pneumococcal bacteremia was associated with CVEs in adults hospitalized with pneumococcal CAP, it was documented that pneumococcal bacteremic CAP was associated with a higher rate of CVEs compared with non-bacteremic pneumococcal CAP, driven primarily by higher rates of new-onset arrythmias 86 (9.3%) versus 64 (5.5%); *p* = 0.01 [14]; (iii) in a retrospective study of 1739 adults hospitalized with pneumococcal CAP, ACE occurred in 304 (17.5%) with older age, pre-existing heart conditions, pneumococcal bacteremia, septic shock on admission, and high-risk pneumonia identified as risk factors [15]; (iv) in a retrospective cohort study of ACE following pneumococcal infection, in which the patients were matched with non-infected patients for each CVE, pneumococcal infections were associated with an elevated risk of subsequent stroke and atrial fibrillation, while effects on short-term health also increased mid-term to long-term susceptibility to CVEs [16]; and (v) in a prospective, multicenter cohort study in hospitalized adults with CAP, age, smoking, chronic heart disease, initial severity of infection, and pneumococcal infections were risk factors for both early and late CVEs and were associated with increased mortality risk during the diagnosis of CAP and after the episode of pneumonia [17]. One recent study reviewed the data that underline the damage that occurs during acute pneumococcal infection that can result in long-term sequelae, including reduction in quality of life and life expectancy in pneumococcal disease survivors [18]. While one earlier study [15] documented no apparent relationship between serotypes or clonal complexes and ACE, a more recent study suggested that serotypes 3 and 9 are more commonly associated with major adverse cardiac events [19].

The two main putative mechanisms that have been implicated in the pathogenesis of myocardial injury in severe pneumococcal pneumonia are the cardiotoxic effects of the pneumococcal pore-forming cytolysin, pneumolysin (Ply), and other pneumococcus-derived products, as well as pro-thrombotic effects involving several pneumococcal virulence factors, including Ply [18,20]. Use of agents active against Ply, or prevention of pneumococcal infections with vaccination, may be strategies to further reduce the impact of pneumococcal CAP and its associated CVEs.

While there need to be studies regarding the potential benefits of influenza and pneumococcal vaccination (PV) against CVEs occurring in patients with influenza and pneumococcal infections, vaccination of patients with underlying cardiac disease could be cost-effective in improving clinical outcomes in those patients [21]. There is evidence for influenza vaccines in protecting against mortality, ACS, and hospitalization in patients with coronary heart disease and CHF; however, the data for PV are generally contradictory. A recent systematic review and meta-analysis to assess the association between recipients of PV and mortality/CV outcomes among patients with and without established CVD documented that PV decreased risk of all-cause mortality and myocardial infarction [22]. Clearly, future randomized controlled trials are needed regarding benefit of PV against CVE and mortality in patients with underlying cardiac disease who have CAP [22]. In this context, it is noteworthy that persons afflicted with sickle cell disease experience an unusually high mortality rate following development of IPD, which is associated with an increased level of sensitivity to Ply [23]. Although it would be interesting to monitor the CVE-associated mortality rates of adults with sickle cell disease following development of IPD, this issue is complicated by the high frequency of age-related, pre-existing CV complications in this setting [24].

## 4. Involvement of Pneumolysin-Mediated Immunosuppression and Cytotoxicity in Disease Pathogenesis

The development of IPD is triggered by the translocation of this dangerous respiratory pathogen from the nasopharynx to the lower airways. Those with co-existent immunosuppression, particularly the non-immunized very young and elderly, are at highest risk. In the very early stages of infection, the pneumococcal pore-forming toxin, Ply, plays a key role in disease pathogenesis by subduing pulmonary host defenses and promoting persistence of the pathogen [25].

### 4.1. Suppression of Alveolar Macrophage Function by Pneumolysin

In the lower airways, the pneumococcus encounters alveolar macrophages, which have a hybrid M1/M2 phenotype, possibly enabling phenotype switching according to changing inflammatory environments [26]. Studies using isolated human or animal macrophages, as well as murine models of experimental infection, have revealed several mechanisms by which the pathogen promotes immunosuppression necessary for early persistence in the lung. This is likely to be most prevalent in non-immune persons in whom antibody/complement-mediated phagocytosis and macrophage activation is minimal. In this setting of early disease, there is significant dependence on pathogen/macrophage scavenger receptor interactions to mediate pathogen clearance, especially the involvement of the C-type lectin, mannose receptor C-type 1 (MRC-1) [27]. Notably, however, adhesion of Ply-expressing wild-type serotype 4 TR4 and serotype 2 D39 strains of the pneumococcus, but not their corresponding isogenic *ply* gene knockout mutants, to isolated human dendritic cells (DCs) and macrophages resulted in detrimental, as opposed to protective, consequences for anti-pneumococcal host defenses. These included attenuation of production of the protective, pro-inflammatory cytokines, tumor necrosis factor (TNF)-α, interleukin (IL)-1β, IL-6, and IL-12 [28]. Mechanistically, Ply-mediated suppression of the early innate, protective, pro-inflammatory cytokine response appeared to involve induction of the suppressor of cytokine signaling 1 (SOCS1), with resultant attenuation of expression of pro-inflammatory genes driven by the transcription factors STAT1 (signal transducer and activation of transcription 1) and NFκB (nuclear factor kappa B) [28]. Dysregulation of cytokine synthesis by Ply also resulted in the induction of naïve T cells with an immunoregulatory phenotype, as well as decreased neutrophil accumulation in the lower airways [28]. The immunosuppressive interaction of Ply with MRC-1 expressed on alveolar macrophages was confirmed in a murine model of experimental infection of the lower airways in which MRC-1-deficient mice exhibited a significant reduction in bacterial loads.

Although these immunosuppressive effects of Ply/MRC-1 interactions are mediated by extracellular pneumococci, it is noteworthy that this mechanism also enables the pathogen to penetrate MRC-1 expressing M2-type macrophages and DCs concealed in MRC-1-coated endosomes. However, these do not fuse with phagosomes, enabling evasion of innate bactericidal mechanisms, which include exposure to reactive oxygen species (ROS), antimicrobial peptides, and proteolytic enzymes, as well as phagosomal acidification, resulting in intracellular survival and persistence of the pathogen. 

Very recently, other investigators using isolated human monocyte-derived macrophages and a murine model of experimental lung infection reported very similar findings [29]. These authors observed that exposure of macrophages to three different Ply-expressing wild-type strains of the pneumococcus (serotypes 4 (TIGR4), 3 and 23F) was significantly less effective than exposure to their Ply-deficient variants with respect to both production of the key protective cytokines IL-1β, IL-6, and TNF-α and control of experimental infection of the lower airways [29]. These immunosuppressive effects of the Ply-expressing strains of the pneumococcus, seemingly mediated via interaction of the toxin with MRC-1, were confirmed by transcriptomic analysis of pathogen-exposed macrophages. Mechanistically, the immunosuppressive activity of Ply was also found to be associated with induction of SOCS1, resulting in inhibition of translocation of NFκB to the cell nucleus and resultant attenuation of the transcription of pro-inflammatory genes [29].

Age-related loss of efficacy of Ply-triggered macrophage LC3-associated phagocytosis represents an additional mechanism, which may contribute to the vulnerability of the elderly for development of severe pneumococcal disease [30]. In this context, isolated murine bone-marrow-derived macrophages exposed to Ply-expressing *S. pneumoniae* undergo an autophagy-independent process of post-phagocytic pathogen containment known as microbutule-associated protein 1A/1B light chain = 3 (LC3)-associated phagocytosis (LAP), in which LC3 is conjugated with the phagosomal membrane to form so-called “LAPosomes” [30]. Unlike autophagosomes, which have a double membrane structure, LAPosomes have a single membrane modulated by ROS, which regulates the internal pH and oxidative state, possibly contributing to pathogen eradication [30,31]. Nevertheless, the exact mechanism of elimination of LAP-targeted bacterial pathogens remains to be established, as does the exact involvement of Ply in their formation. Notably, however, macrophages from aging mice have been found to have an impairment of LC-3-associated phagocytosis, manifested as defective control of pneumococcal infection. Although of potential importance, the clinical relevance of these findings remains uncertain. 

Interestingly, exposure of isolated human monocyte-derived macrophages to Ply has been linked to epigenetic changes in these cells, which are also associated with early immunosuppression [32]. The design of this study involved infecting the cells with either the wild-type D39 strain of *S. pneumoniae*, or the corresponding *ply* gene-deficient mutant, or the reconstituted mutant [32]. Following initiation of experimental infection, the authors monitored the resultant transcriptomic, proteomic, and epigenetic landscapes of the macrophages. As described by others [28,29], they demonstrated early differential inflammatory gene expression, which was significantly attenuated in macrophages exposed to the two Ply-replete strains of the pathogen, according to decreased production of TNF-α and IL-6 [32]. In addition, quantitative mass spectrometry revealed extensive histone post-translational modifications, encompassing 94 histone peptide proteoforms, 5 of which were altered in a Ply-dependent fashion (3 increased, 2 decreased). Although possible associations of these with Ply-mediated attenuation of the innate immune response remain to be conclusively established, it is noteworthy that inclusion of the histone deacetylase inhibitor, vorinostat, mimicked the suppressive effects of Ply on macrophage production of TNF-α [32].

### 4.2. Dendritic Cells

As alluded to above, exposure of isolated monocyte-derived DCs to a Ply-expressing strain of *S. pneumoniae* resulted in attenuation of production of immunostimulatory cytokines and failure to activate CD^4+^ T cells [28]. More recently, Paulikat et al. have confirmed and extended these findings [33]. Like Subramanian et al. [28], these authors compared the expression of biomarkers of immune activation following exposure of human, cytokine-differentiated monocyte-derived DCs to wild-type or *ply* gene-knockout strains of the TIGR4 strain of *S. pneumoniae* [33]. They observed that relative to the responses of the Ply-deficient variant of the pathogen, exposure of DCs to the wild-type strain resulted in defective maturation of these cells. This was characterized by attenuation of production of the pro-inflammatory cytokines, IL-6, IL-12, and IL-23, as well as decreased expression of the pro-adhesive, co-stimulatory immune checkpoint molecules CD40, CD80, and CD86 [33]. In addition, dysregulation of DC activation by Ply resulted in failure of these cells to activate CD4^+^ T cells and possibly, but not proven, induction of Th17 cells [33]. The authors also observed that Ply-mediated suppression of the reactivity of DCs was augmented by co-infection with influenza A virus, in keeping with the heightened threat posed by simultaneous infection with these dangerous respiratory pathogens [33]. 

This section of the review has highlighted the vulnerability of alveolar macrophages, as well as DCs, that populate the lower airways, to Ply-mediated early immune dysfunction. These are clearly key events in the pathogenesis of severe pneumococcal disease, which result in early failure to control the infection in susceptible individuals. In this setting of early induction of Ply-mediated immunosuppression, cell wall-associated toxin, as opposed to that released extracellularly by the action of LytA-mediated autolysis during the late stages of bacterial growth, may also play a significant role [34,35]. As shown in murine models of experimental infection of lower airways, significant growth of Ply-expressing strains of the pathogen occurs during the 24 h period post induction of infection with the rate of bacterial growth in the lungs dependent on the capsular serotype of the pathogen [36]. Synthesis of Ply occurs during the logarithmic phase of growth and the toxin, which lacks an N-terminal secretion signal sequence, is released extracellularly by autolysin-mediated disintegration of the peptidoglycan backbone of the pathogen, significantly increasing the threat of cytotoxicity and pulmonary damage, which favor extra-pulmonary dissemination of the pneumococcus. At these high concentrations of Ply, the cholesterol-binding pore-forming activity of the toxin is prevalent, with the potential to damage and kill multiple cell types, not only by necrosis, but also by triggering programmed cell death pathways such as apoptosis and necroptosis, depending on cell type [37,38,39].

## 5. Involvement of Pneumolysin in Extra-Pulmonary Dissemination of the Pneumococcus

The mechanisms involved in the pathophysiology of Ply-mediated acute lung injury in the experimental setting, which favor extracellular dissemination of the pneumococcus, have been extensively investigated by Witzenrath et al. [40]. Using isolated, ventilated and blood-free murine lungs, these authors explored the effects of a 30 min challenge with recombinant Ply (0.025–2.5 micrograms (μg)), administered either by infusion into the pulmonary artery or intratracheally using aerosolized toxin, on lung microvascular permeability and pulmonary vascular resistance [40]. Administration of Ply by both routes caused significant, dose-related increases in capillary permeability, while only infusion of the toxin resulted in significantly increased pulmonary vascular resistance. Immunohistochemistry revealed that Ply was present predominantly in the endothelial cells of arterial vessels, which manifested robust vasoconstriction [40]. Additional in vitro experiments revealed that exposure of an alveolar epithelial cell line (A549) and human umbilical vascular endothelial cell monolayers to Ply (0.1–1 μg) resulted in significantly increased permeability of both cell types, which was maximal after 60 min of incubation, while intercellular gap formation was evident in the endothelial cell monolayers [40]. This study indicates that acute pneumococcal pneumonia results in Ply-mediated disruption of alveolocapillary barrier function and pulmonary hemodynamics, not only causing respiratory failure, but also enabling extrapulmonary dissemination of both the pathogen and its pore-forming toxin. 

A combination of mechanisms triggered by the pore-forming interactions of Ply with various types of resident and migratory cells are likely to underpin the pathogenesis of pulmonary damage and dysfunction in severe pneumococcal disease. These include:

- Most prominently, the direct cytotoxic effects of Ply on alveolar epithelial and endothelial cells, resulting from necrosis and necroptosis [41,42]. In this context, the mechanism of necroptotic cell death is independent of the death receptor and is achieved by the initiation of signaling events triggered by Ply-mediated calcium influx and potassium efflux [42,43];
- Induction of the synthesis of platelet-activating factor (PAF) in lung tissue exposed to the toxin, as reported by Witzenrath et al., in a follow-up study [44]. Although the cellular origin of PAF was not identified, interactions of this bioactive lipid with endothelial cells was proposed to induce the synthesis of the potent vasoconstrictor, thromboxane A2 [44];
- Pore formation due to exposure of endothelial cells to lower, sub-lytic concentrations of Ply, triggering calcium influx, which causes “adherens function disassembly and cytoskeletal rearrangement to facilitate endothelial cell retraction and increased permeability” [45];
- Involvement of the regulator of endothelial permeability, angiopoietin-2 (ang-2), in Ply-induced endothelial permeability, as reported more recently by Gutbier and other members of the Witzenrath research team, using the isolated murine perfused and ventilated lung model, as well as other approaches [46]. These authors reported the following: (i) perfused and ventilated lungs from ang-2 gene-knockout mice demonstrated reduced permeability on exposure to Ply; (ii) exposure of three different types of endothelial cell (human pulmonary microvascular; human umbilical vein; murine lung) to Ply (1–2 μg/mL) in vitro resulted in dose-related increased production of ang-2, while endothelial barrier integrity was impaired following exposure of the endothelial cells to ang-2; (iii) elevated serum concentrations of ang-2 had negative prognostic value in patients with CAP; and (iv) ang-1, an antagonist of ang-2, which inhibits calcium influx [47], attenuated Ply-mediated augmentation of endothelial permeability, implicating sub-lytic pore formation and calcium influx in toxin-mediated synthesis of ang-2 [46];
- Platelet activation per se, as well as platelet-driven activation of vascular endothelium and infiltrating neutrophils, represent additional contributors to the pulmonary damage and dysfunction during acute pneumococcal pneumonia, which presumably precedes, and appears to be essential for subsequent, cardiac injury. In this context, several inter-linked developments are of particular note. Firstly, the discovery by Le Français et al. that the lungs of mice and humans represent a primary site of thrombopoiesis, with the pulmonary circulation of mice accounting for approximately 50% of the body pool of platelets in mice [48], with corresponding estimates of platelet production in the lungs of humans and mice of 7–100% [49]. Secondly, Ply has been reported to induce platelet activation in several [50,51,52], but not all [53], studies, and to promote the formation of neutrophil:platelet aggregates [54] in vitro. If operative in the setting of acute pneumococcal pneumonia, these mechanisms are likely to trigger endothelial activation and pulmonary microvascular injury and occlusion. In addition, Ply has been shown to activate the formation of neutrophil extracellular traps (NETs) by isolated human neutrophils [51,55]. Notably, platelet-driven NETosis is a recognized driver of intravascular coagulation and thrombin activation during severe microbial and viral infections [56,57]. It has also been implicated in the pathogenesis of the pulmonary complications of severe COVID-19 [58,59], as well as in the severity of experimental pneumonia in mice [60]. These mechanisms are probable key contributors to the pathogenesis of IPD in humans.

Acting in concert, the aforementioned Ply-mediated mechanisms, which are summarized in Table 1, are likely to inflict the mechanical damage to the lungs, which precedes and is a prerequisite for extrapulmonary dissemination of the pneumococcus, setting the scene for invasion of distal organs. Although alveolar epithelial cells and endothelial cells possess several mechanisms which are mobilized to repair Ply-mediated pores, these mechanisms vary with respect to efficacy and cell type [61,62,63,64]. In the case of myeloid cells, pore-damaged membrane is expelled via formation of calcium-dependent vesicles [61,62], while alveolar cells and endothelial cells utilize attenuation of the activities of executioner caspases 3/7 and induction of caveolin-1-mediated endocytosis-dependent mechanisms, respectively [63,64].

## 6. Role of Pneumolysin in Cardiac Dysfunction during Severe Pneumococcal Disease

Of the common bacterial CAP pathogens, the pneumococcus appears to be the most cardiotropic and cardiotoxic due to the pore-forming cytolytic potency of Ply. Of the other CAP pathogens, *Staphylococcus aureus*, albeit a relatively uncommon cause of CAP, produces a pore-forming cytolysin, α-hemolysin. This bacterial toxin initiates lethal membrane damage via a complex mechanism following interaction with sphingomyelin/cholesterol [65], which has been implicated in the pathogenesis of coronary dysfunction in a murine model of experimental cardiotoxicity [66].

Severe pulmonary damage inflicted by Ply, acting in concert with other pneumococcal toxins, especially hydrogen peroxide, enables escape and systemic spread of the pathogen. Invasion of the myocardium, for which the pneumococcus has a notable predilection, represents a significant risk, particularly during the first seven days of development of IPD when the incidence rate ratio peaks at 92.95 (95% CI; 72.17–119.71) [67]. The involvement of Ply in the pathogenesis of IPD-associated CVEs has been expertly and convincingly explored in a series of murine and non-human primate experimental infection studies [68,69,70], which have been collated in several reviews [20,25].

These studies revealed that the initial event in translocation of the pneumococcus to the myocardium is strain-specific and dependent on a high degree of bacteremia [71] and possibly on the level of expression of the ZomB protein, which is a novel positive transcriptional regulator of the *ply* operon [72]. The interaction of the bacterial adhesins, choline-binding protein A (CbpA), and phosphorylcholine with the endothelium of myocardial microcapillaries are key events, which enable translocation of the pathogen from the circulation into the heart tissue [68,69]. The pneumococcus then becomes established in early microlesions, perpetuating its presence via Ply-mediated necroptosis of infiltrating and resident, sentinel cardiac macrophages [73,74]. The resultant immunosuppression enables the formation of mature, persistent intra-cardiac microlesions and transition of the pneumococcus to a biofilm-forming/high Ply-producing phenotype [74]. These events, in turn, inflict impaired cardiomyocyte contractibility and cell death, events which precede severe myocardial dysfunction [75]. Furthermore, it has been demonstrated in these animal models that successful treatment of the pneumococcal infection, with the use of appropriate antibiotics, is associated with maturing of the microlesions, with immune cell infiltration and collagen deposition, suggesting that there is long-term cardiac scarring, which may well explain the occurrence of cardiac complications, such as cardiac arrhythmias, even some time after the acute infection [68].

Importantly, a recent experimental animal study has highlighted the risk posed by co-infection with influenza A virus (IAV) and IPD on the incidence and severity of cardiac damage and dysfunction. In this study, Platt et al. investigated the effects of induction of infection of mice with a mouse-adapted strain of IAV followed 10 days later by challenge with the TIGR4 strain of the pneumococcus [76]. Co-infection with both respiratory pathogens, as opposed to the pneumococcus alone, resulted in a significant increase in the percentage area of the heart, which manifested demonstrable microlesions, as well as a 1000-fold increase in bacterial titers [76]. Mechanistically, primary infection with IAV resulted in significant upregulation of cardiomyocyte adhesion molecules, specifically the polymeric Ig receptor and fibronectin leucine-rich transmembrane protein 1, which interact with the pneumococcal adhesins PspC and PfbA/PavA/PavB, respectively [75]. Complementary in vitro studies revealed that exposure of AC16 cardiomyocytes to the wild-type strain of the pneumococcus resulted in high-level cytotoxicity, which was attenuated by selective knockout of any one of the genes encoding the pneumococcal adhesins, PspA, CbpA, or PsrP, as well as that encoding Ply [76]. The involvement of Ply in co-infection-mediated augmentation of cardiotoxicity was strengthened by the observation that pre-treatment of cardiomyocytes with recombinant toxin significantly increased the degree of cellular injury [76].

As with pulmonary damage, a second, possibly interactive, albeit less intensively explored, mechanism of cardiotoxicity, relates to the potential of Ply to promote systemic activation of platelets and neutrophils. These events may result in myocardial microvascular occlusion due to the formation of platelet:platelet homotypic aggregation and platelet:neutrophil heterotypic aggregation, as well as platelet-driven NETosis [20,25].

A third potential mechanism of Ply-mediated cardiotoxicity, albeit unexplored in the setting of experimental pneumococcal infection, involves the interaction of Ply and pneumococcal neuraminidase A (NanA) with high-density lipoproteins [77]. In this study, Syed et al. observed that exposure of HDL particles to the combination of Ply and NanA in vitro resulted in significant alterations to the sialic acid, protein, and lipid composition of the lipoproteins. These alterations in molecular structure were associated with oxidative damage and pro-inflammatory activity, as well as increased complement activity, resulting in increased pro-atherogenic potential [77].

These various mechanisms of Ply-mediated cardiotoxicity are summarized in Table 2.

The preceding sections of this review, which have focused on Ply, clearly identify this toxin as a key target in the therapy of IPD and protection against development of CV complications. In this context, and in keeping with the recent clinical study by Chowers et al., we focused on the role of the type of antimicrobial chemotherapy in mortality in bacteremic pneumococcal pneumonia, reinforcing the role of macrolide antibiotics in protecting against Ply, which is considered in the following sections [78].

## 7. Antibiotic Treatment

Regarding antibiotic treatment of CAP, current guidelines indicate that despite concerns of increasing antibiotic resistance among the pathogens, most patients with CAP can currently still be successfully treated with antibiotic regimens that have been used for many decades [79]. One area of particular concern with regard to antibiotic resistance has been macrolide resistance, which has been shown to be associated with variable, though generally high, rates of resistance among pneumococcal isolates in the United States, such that some authors have suggested that there should be reconsideration of macrolide monotherapy, at least in some areas of the country [80]. Another area of controversy with regard to macrolide use is the need for beta-lactam/macrolide (BM) combination therapy in outpatients with comorbidities, or inpatients with risk factors for antibiotic resistant pathogens, or severe CAP, versus the use of fluoroquinolones (FQs) alone, as per current guidelines [79,81,82]. Cilloniz et al. conducted a retrospective, observational study of adults hospitalized for CAP who had positive cultures for *S. pneumoniae*, and found no evidence that patients infected with macrolide-resistant pathogens were either more severely ill or had worse outcomes if treated with guideline compliant therapy [83]. Despite increasing resistance, based on the evidence from the Cilloniz et al. study, as well as other studies, many authors continue to recommend the use of macrolides, usually as part of combination therapy, because of their non-antimicrobial anti-inflammatory effects [81,82,84]. Authors have also suggested that patients with COVID-19 pneumonia and suspected co-infection with CAP, especially if severe, should receive BM therapy as for CAP alone [85,86].

## 8. Benefits of Beta-Lactam/Macrolide Therapy in Patients with CAP

Several more recent studies have evaluated the potential benefits of BM combination therapy in patients with CAP, compared with beta-lactam monotherapy (BL) or FQ monotherapy.

### 8.1. Non-ICU Hospitalized CAP

A systematic review and meta-analysis, as well as a network meta-analysis, indicated that FQ monotherapy had similar efficacy and safety compared with BL with or without a macrolide [87,88]. Furthermore, in a retrospective cohort study there was no difference in 30-day readmissions in patients with CAP who received FQ versus BM [89]. In contrast, in a retrospective cohort study in Korea, BM combination therapy among patients with a CURB-65 score ≥ 2, the 30-day survival rate was higher in the BM combination than in the BL monotherapy-treated group (93.3% versus 88.5%; *p* = 0.009), but only in autumn [90]. In a cohort of patients with CAP of moderate severity (non-ICU cases), a simple decision tree distinguished patients who would benefit from the addition of a macrolide to a BL agent, leading to a 27% (OR 1.83, 95% CI 1.48–2.27; *p* < 0.001) reduction in mortality, compared to standard of care [91]. This outcome benefit needs a randomized controlled trial to confirm the findings.

### 8.2. Moderate-to-Severe CAP, including ICU Patients

Three studies evaluated combination therapy versus BL or FQ monotherapy. The first showed that the efficacy of ceftriaxone combination therapy was similar to that of FQ monotherapy, as well as being associated with lower drug-related adverse events [92]. The second study demonstrated no differences in short- or long-term outcomes in CAP patients treated with either BM combination therapy or FQ monotherapy [93]. The third study showed that compared to BL monotherapy, treatment with azithromycin plus BL had no benefit on the in-hospital mortality of mechanically ventilated patients with CAP and CAP-associated ARDS [94].

In contrast to the former studies, several additional studies have shown the benefit of BM combination antibiotic therapy over other therapies in hospitalized cases with CAP, including those admitted to ICU. One prospective, observational study of hospitalized cases with CAP of known microbial cause indicated that BM combination therapy was associated with decreased mortality compared with FQ monotherapy or FQ in combination with a BL in pneumococcal CAP and in patients with a higher systemic inflammatory response (CRP > 15 mg/dL) [95]. When both factors were present, BM was protective against mortality on multivariate analysis [95]. No benefit was seen in those without a high inflammatory response and pneumococcal CAP, or in those with other etiologies of CAP. In a more recent retrospective, observational study of patients with pneumococcal CAP, patients with bacteremia were found to have a higher 30-day and 1-year all-cause mortality compared to those with negative blood cultures (26.2% versus 16.3%; age adjusted hazard ratio (aHR) 1.84; 95% CI 1.33–2.53 and 38.5% versus 30.4%; aHR 1.43; 95% CI 1.05–1.96, respectively) [96]. There was both a 30-day and 1-year mortality benefit in patients receiving combination therapy with a macrolide, but only for those patients with bacteremia, not for those with negative blood cultures. In a prospective cohort study, BL therapy was compared with BM therapy (with use of azithromycin) in non-ICU cases hospitalized with CAP [97]. Based on the CURB-65 and Pneumonia Severity Index scores, combination therapy did not significantly reduce 30-day mortality in either group. However, based on Infectious Diseases Society of America/American Thoracic Society criteria, combination therapy significantly reduced 30-day mortality in patients with severe, but not non-severe, CAP (odds ratio (OR) 0.12; 95% CI 0.007–0.57 versus OR 1.85; 95% CI 0.51–5.40).

One single-center, retrospective cohort study in patients with severe CAP indicated that in those who had a negative BioFire PCR result, de-escalation from BM combination therapy was not associated with increased in-hospital mortality [98]. However, this fits in well with a more recent study of 2016 cases with bacteremic pneumococcal pneumonia, in which 1921 were treated with empiric antibiotics with anti-pneumococcal coverage (ceftriaxone) and 1159 received “coverage for atypical pathogens” (macrolides or a fluoroquinolone) [78]. Independent predictors of mortality were age, being at high risk of pneumococcal disease, and multilobar pneumonia, while female sex and receipt of macrolide therapy were protective (OR 0.708; 95% CI 0.522–0.960 and OR 0.549; 95% CI 0.391–0.771, respectively). Treatment with either azithromycin or roxithromycin for as little as two days was protective and a full course of therapy was not required.

### 8.3. Conclusions Regarding the Clinical Benefits of Combination Therapy

As indicated in the more recent studies described above, as well as in a review of the literature by Shumilak et al. [99], the bulk of the current evidence suggests that in adults hospitalized with CAP, BL monotherapy was equivalent to combination therapy in cases that were mild to moderate in severity, but that severe cases managed with BL monotherapy had worse clinical outcomes when compared with combination therapy. However, many of the authors of the publications described above, from which the conclusions were drawn, indicate that because of the limitation in quality and quantity of the studies, more randomized controlled trials with large numbers and high quality need to be performed to verify the findings [87,88,99,100,101].

Regarding additional mechanisms by which macrolides may attenuate cardiac events in severe CAP, it is interesting to note that in one pre-clinical trial, azithromycin and clarithromycin have been noted to possibly antagonize PAF [102]. One hypothesis-generating study was undertaken in patients with severe pneumonia (as defined by the IDSA/ATS guidelines) to determine if patients receiving a macrolide together with acetylsalicyclic acid (ASA), in addition to conventional antibiotics, would derive further benefit than those receiving conventional antibiotic therapy alone. Patients were divided into four groups: (i) those receiving only ASA, (ii) those receiving macrolides only, (iii) those receiving ASA plus macrolide, and (iv) those receiving neither ASA nor macrolide [103]. The mortality at 28 days was 15.5% in the ASA-macrolide group versus 28.2% in the group receiving neither agent and 23.8% in the macrolide group and 21.1% in the ASA group. After propensity score analysis, receipt of ASA plus a macrolide (HR 0.71; 95% CI 0.58–0.88; *p* = 0.002) was associated with a higher 30-day survival rate.

## 9. Are the Benefits of Combination Antibiotic Therapy Specifically Due to the Macrolide?

Two systematic reviews and meta-analyses compared the relative benefits of BM combination therapy with use of a BL plus FQ combination therapy (BLFQ) [100,101]. The authors indicated that one could not elicit strong conclusions on the findings because of high risk of bias, methodological issues, and low quality of evidence in the various trials. The former study indicated that in the preliminary analysis, mortality of the BLFQ combination was higher than for BM combination therapy, but that in the meta-analysis with adjusted mortality rates there was no significant benefit of either of the two therapies (RR 1.26; 95% CI 0.95–1.67). However, the latter study in patients with severe CAP indicated that there was a significantly lower mortality in the BM versus the BLFQ therapy group and the length of hospital, but not ICU, stay was also lower. It is important to recognize that inter-class macrolide differences have been noted in some studies. While one systematic review and meta-analysis found no comparator studies between erythromycin and azithromycin, studies comparing erythromycin with clarithromycin showed erythromycin to be less effective [104]. Furthermore, a systematic review of azithromycin versus clarithromycin in combination with a BL, showed a higher success rate for azithromycin-based combinations, but a shorter hospital length of stay with clarithromycin-based combinations [105].

The reason for the differences in benefits of macrolide versus FQ combination therapy, if there are any, is not due to differences in efficacy against “atypical bacteria” as indicated in a recent systematic review and meta-analysis, which showed no difference in rates of clinical failure of CAP due to these pathogens [106]. It is also interesting that a recent retrospective study confirmed what other authors have previously shown, that the benefit of adding azithromycin to the antibiotic regimen in patients in ICU with pneumococcal CAP is significantly associated with a reduction in in-hospital mortality, independent of multiple co-variates even in the presence of macrolide resistance [107]. Furthermore, and rather interestingly, in a secondary analysis of the CAPO Internal Cohort study data, patients were grouped according to the sequence of administration of the macrolide and BL [108]. Overall, 99 patients were included in the “macrolide before” group and 305 in the “macrolide after” group. Administration of a macrolide before compared to after the BL reduced time to clinical stability (3 versus 4 days; *p* = 0.011), length of stay (6 versus 7 days; *p* = 0.002), and mortality 3% versus 7.2%; *p* = 0.229). Recent studies on the benefits of beta-lactam/macrolide combination therapy on outcome in adults hospitalized for CAP are summarized in Table 3.

In addition to their antimicrobial activities, especially inhibitory effects on the production of Ply, it is noteworthy that macrolides specifically are associated with a myriad of non-antimicrobial actions that have been reviewed elsewhere [84].

## 10. Macrolide Antibiotics as Regulators of Pneumolysin Production

Many strategies, which target the reactivity of Ply have been described and represent the topic of several recent reviews [25,39,109]. These include a number of small molecules of natural and synthetic origin, monoclonal antibodies, liposome-encased cholesterol, and vaccine-based strategies. Despite showing efficacy in murine models of experimental pneumococcal infection, these strategies, with the exception of cholesterol-containing liposomes [110], are, however, of unproven clinical efficacy. On a more positive note, innovative approaches to the development of pneumococcal protein-targeted vaccines show promise [111], as do competitive, synthetic peptides based on the Ply-binding site of the mannose receptor, MRC-1 [112].

Currently, however, inhibitors of Ply production, specifically macrolide antibiotics, appear to represent the most effective, inexpensive adjunctive option in augmenting the BL antibiotic-based treatment of IPD and presumably prevention of CVEs. In this context, the potential of macrolides, at sub-minimum inhibitory concentrations (MICs), to inhibit the production of Ply, by not only macrolide-susceptible, but also macrolide-resistant strains of the pathogen, has been known for over twenty years [113,114,115,116].

Macrolides are primarily inhibitors of bacterial protein synthesis, which bind to the peptide exit tunnel of the bacterial 50S ribosome, resulting in interference with peptide chain elongation [117]. Most commonly, macrolide resistance (≥256 μg/mL) results from acquisition by the pneumococcus of the inducible *erm*(B) gene, which encodes the enzyme ribosomal methylase. This enzyme dimethylates a single adenine residue in 23S ribosomal RNA, leading to high-level macrolide resistance. With respect to mechanisms by which macrolide-mediated attenuation of production of Ply is maintained in the setting of *erm*(B) positivity, Cockeran et al. reported that effective induction of *erm*(B) following exposure of strain 2507 of the pneumococcus (serotype 23F; MIC ≥ 256 μg/mL) to 0.5 μg/mL clarithromycin had a slow onset of approximately 12.5 h [118]. This, in turn, resulted in transient susceptibility to clarithromycin and other macrolides, causing inhibition of both growth and production of Ply [118]. This mechanism was also detected in seven other *erm*(B)-expressing strains of the pneumococcus, albeit with variable lag times (4.9–11.5 h) until full expression of the resistance phenotype. In addition, sequential exposure of strain 2507 to clarithromycin (0.5 μg/mL) followed 45 min later by ceftriaxone (0.05–0.5 μg/mL) resulted in significant, synergistic inhibition of bacterial growth [118].

More recently, Domon et al. described additional mechanisms of macrolide-mediated inhibition of Ply synthesis and release following exposure of strain NU4471 of the pneumococcus (serotype 19; MIC ≥ 1000 μg/mL) to azithromycin, clarithromycin or erythromycin (2 and 4 μg/mL) [119,120]. In their initial study, focused on azithromycin and erythromycin, these authors reported that both macrolides, particularly erythromycin, caused inhibition of release of the autolysin, LytA, by the pneumococcus [119]. In addition, both macrolides caused significant downregulation of transcription of the *ply* gene, while transcription of *lytA* was unaffected [119]. In a follow-up study, in which they compared clarithromycin with erythromycin, the dual mechanism of erythromycin-mediated inhibition of Ply synthesis and release was confirmed, while exposure to clarithromycin caused significant inhibition of transcription of the *ply* gene in the setting of potentiation of autolysis [120]. The findings of these two studies reported by Domon et al. appear to indicate that inhibition of transcription of the *ply* gene is a mechanism common to macrolide antibiotics, while effects on autolysis are variable [119,120].

## 11. Conclusions

There remains a considerable global burden of CAP, and in most areas of the world, *S. pneumoniae* remains the predominant pathogen. It is now well recognized that CVEs are a relatively common occurrence in patients hospitalized with CAP and that the pneumococcal virulence factor, Ply, plays a major direct role in these cardiac events, as well as having additional significant pro-inflammatory activities, which contribute, albeit indirectly, to these events. Regarding antibiotic therapy, it is generally accepted that the use of combined beta-lactam and macrolide therapy is associated with better outcomes in severely ill patients with CAP. In addition to their antibiotic effects, macrolides are well known to have anti-inflammatory, immunomodulatory activity, including significant ability to attenuate the production and activity of Ply, even in the setting of macrolide resistance. Notwithstanding prior immunization of the vulnerable elderly against both the pneumococcus and influenza virus, adjunctive Ply-targeted therapeutic strategies to support beta-lactam/macrolide combination therapy include the administration of cholesterol-encased liposomes and, albeit untested in the clinical setting, early administration of synthetic, diversionary MRC-1 peptide mimetics.

## Figures and Tables

**Table 1:** Pneumolysin-mediated mechanisms of immunosuppression and endothelial barrier disruption operative in the lower respiratory tract, which promote proliferation and extra-pulmonary dissemination of the pneumococcus.

Mechanisms of Immunosuppression *	Mechanisms of Endothelial Barrier Disruption **
Suppression of the protective pro-inflammatory activity of alveolar macrophages and dendritic cells via interaction of Ply with MRC-1 and induction of SOCS1	Direct cytotoxic effects on alveolar epithelial and endothelial cells due to the pore-forming actions of the toxin
Age-related impairment of LC-3-associated phagocytosis	Induction of synthesis of PAF, which, in turn, activates the synthesis of thromboxane A2 by vascular endothelial cells
Induction of Ply-associated epigenetic changes following exposure of macrophages to the pneumococcus associated with decreased production of protective, pro-inflammatory cytokines	Upregulation of production of the regulator of vascular permeability, angiopoietin-2, by endothelial cells, which, in turn, causes impairment of endothelial barrier function
Defective maturation of dendritic cells associated with decreased production of both pro-inflammatory cytokines and expression of co-stimulatory immune checkpoints, as well as failure to activate CD^4+^ T cells	Induction of platelet activation leading to pulmonary microvascular injury and occlusion due to intravascular formation of platelet:platelet homotypic aggregates, platelet:neutrophil aggregates, and platelet-driven NETosis

* References [28,29,30,31,32,33]; ** References [40,41,42,43,44,45,46,47,48,49,50,51,52,53,54,55,56,57,58,59,60]; Abbreviations: MRC-1 (mannose receptor C-type 1); SOCS1 (suppressor of cytokine signaling 1); LC3 (microtubule-associated protein 1A/1B-light chain 3); Ply (pneumolysin); PAF (platelet-activating factor); NETs (neutrophil extracellular traps).

**Table 2:** Summary of pneumolysin-mediated mechanisms of immunosuppression and cardiotoxicity in the pathogenesis of severe myocardial dysfunction.

Mechanism	References
Ply-mediated suppression of the protective activities of infiltrating and resident sentinel cardiac macrophages via induction of necroptosis following adherence of the pathogen to the vascular endothelium of myocardial capillaries	[68,69,70]
Following invasion of the myocardium the pneumococcus becomes established in intra-cardiac microlesions in which it transitions to a biofilm-forming, high Ply-producing phenotype	[71,73,74]
Cardiotoxicity results from interaction of the pneumococcal adhesins PspA, CbpA, and Psrp with cardiomyocytes, resulting in exposure of these cells to Ply, leading to cell death and myocardial dysfunction	[75,76]

Abbreviations: Ply (pneumolysin); PspA (pneumococcal surface protein A); CbpA (choline-binding protein A); Psrp (pneumococcal serine-rich repeat protein).

**Table 3:** More recent studies on benefits of beta-lactam macrolide combination antibiotic therapy in terms of outcome in adults hospitalized for CAP.

Study, Year	Country	Patient Number	Type of Study	Site of Care	Comparison	Measure of Severity of Illness	Outcomes
Ito et al. 2019 [97]	Japan	1131	Prospective cohort study. Hospitalized cases.	Non-ICU	BL versus BLM	PSI, CURB-65, IDSA/ATS criteria were measured. Patients were classified as mild, moderate, or severe.	Based on PSI and CURB-65 severity scores, combination therapy did not reduce 30-day mortality, in either treatment group. Based on IDSA/ATS criteria for severity, combination therapy significantly reduced 30-day mortality in severe, but not non-severe pneumonia (OR 0.12; 95%CI 0.007–0.57).
Ceccato et al. 2019 [95]	Spain	1715	Prospective observational cohort study. Hospitalized cases of known microbial etiology.	ICU and non-ICU cases	BL plus FQ or FQ alone versus BLM	High inflammatory response (CRP > 15 mg/dL)	BLM had a protective effect on mortality only in cases with a high inflammatory response and pneumococcal CAP (adjusted OR 0.28; 95% CI 0.09–0.93), but not in those without a high inflammatory response and pneumococcal CAP or with other etiologies.
Shorr et al. 2021 [107]	USA	140	Retrospective cohort study of hospitalized patients with pneumococcal pneumonia, including cases with M-resistant pathogens	ICU	Comparison of outcomes in patients treated with antibiotic therapy with or without a M	Markers of acute and chronic disease (e.g., Charlson score, need for mechanical ventilation, and/or vasopressors and APACHE II)	The addition of M to the antibiotic regimen was associated with significant reduction in in-hospital mortality independent of multiple co-variates (adjusted odds ratio of death in those on macrolide 0.27; 95% CI 0.09–0.85; *p* = 0.024).
Goncalves-Pereira et al.2022 [96]	Portugal	797	Prospective multicenter study of hospitalized patients with pneumococcal CAP (bacteremic or non-bacteremic) with at least one comorbidity	ICU and non-ICU	Assessment of the benefit of BLM therapy versus non-BLM therapy in cases with and without bacteremia	-	Patients with bacteremia had higher 30-day all-cause mortality and BLM was beneficial only in patients with bacteremia (30-day all-cause mortality 18.9% versus 36.1%, aHR 0.49; 95% CI 0.30–0.80; *p* = 0.004). After 1-year follow-up, patients with bacteremia who had BLM still had a lower all-cause mortality (31.3% versus 48.1%; *p* = 0.009).
Chowers et al. 2023 [78]	Israel	2016	Part of an ongoing prospective population-based active surveillance study of adult cases with bacteremic pneumococcal pneumonia.	ICU and non-ICU cases	BLM versus other antibiotic therapy with no macrolide	Patients classified as no-risk, at-risk, and high-risk of invasive pneumococcal disease	Macrolide therapy for as short as two days was protective against mortality (OR 0.549; 95% CI 0.391–0.771).

Abbreviations: ICU (intensive care unit); BL (beta-lactam); BLM (beta-lactam/macrolide combination FQ (fluoroquinolone); PSI (pneumonia severity index); CURB-65 (confusion, urea, respiratory rate, blood pressure, age ≥ 65 years); OR (odds ratio); aHR (adjusted hazards ratio).

## Data Availability

Not applicable.
